# The ERUS course on robot-assisted kidney transplantation

**DOI:** 10.1007/s00345-024-04802-y

**Published:** 2024-03-30

**Authors:** Riccardo Campi, Alessio Pecoraro, Federico Piramide, Maria Lucia Gallo, Sergio Serni, Alex Mottrie, Angelo Territo, Karel Decaestecker, Alberto Breda, On behalf of the European Association of Urology (EAU) Robotic Urology Section (ERUS) Robot-assisted Kidney Transplantation working group

**Affiliations:** 1 Unit of Urological Robotic Surgery and Renal Transplantation, Careggi Hospital, Florence, Italy; 2 Department of Experimental and Clinical Medicine, University of Florence, Largo Brambilla, 3, 50134 Florence, Italy; 3 Departement of Urology, Onze-Lieve-Vrouwziekenhuis Hospital, Aalst, Belgium; 4 ORSI Academy, Ghent, Belgium; 5 Division of Urology, Department of Oncology, San Luigi Gonzaga Hospital, University of Turin, Turin, Italy; 6 Department of Urology, Fundaciò Puigvert, Autonomous University of Barcelona, Barcelona, Spain; 7 Department of Urology, ERN eUROGEN Accredited Centre, Ghent University Hospital, Ghent, Belgium; 8 Department of Urology, AZ Maria Middelares Hospital, Ghent, Belgium; 9 Chirurgia Urologica Robotica Mini-Invasiva e dei Trapianti Renali, Azienda Ospedaliero-Universitaria Careggi, Viale San Luca, 50134 Florence, Italy

**Keywords:** Course, Kidney transplantation, Robotics, Simulation, Training

## Abstract

**Purpose:**

Robot-assisted kidney transplantation (RAKT) is being increasingly performed at selected referral institutions worldwide. Yet, surgical training in RAKT is still unstructured and not grounded into formal credentialing courses including simulation, lab facilities, and modular training with animal models. As such, developing standardized, modular training programs is warranted to provide surgeons with the RAKT-specific skillset needed for a “safe” learning curve.

**Methods:**

The 3-day course on RAKT developed at the EAU Skills Center in Orsi Academy was designed as a standardized, modular, step-by-step approach aiming to provide theoretical and practical skills. The course is held by expert proctors with extensive experience in RAKT. To maximize the course’s usefulness, a solid knowledge of robotics and transplantation is desirable for participants.

**Results:**

From January 2016 to July 2023, 87 surgeons from 23 countries (of which 36% from extra-European countries) participated in the RAKT course performed at the EAU Skills Center in Orsi Academy. Of these, 58/87 (67%) were urologists, while 27/87 (31%) were general surgeons and 2/87 (2%) were vascular surgeons. To date, 18 participants (20.6%) are actively involved in RAKT programs at institutions included in the European Association of Urology (EAU) Robotic Urology Section (ERUS)–RAKT network.

**Conclusion:**

Leveraging the potential of simulation, wet-lab training, live porcine models, and experienced proctors, the RAKT course performed at the EAU Skills Center in Orsi Academy represents the first structured teaching effort aiming to offer surgeons a full immersion in RAKT to train the core technical skills.

**Supplementary Information:**

The online version contains supplementary material available at 10.1007/s00345-024-04802-y.

## Introduction

Robot-assisted kidney transplantation (RAKT) is emerging as a safe minimally invasive approach, and it has been increasingly performed at selected referral institutions worldwide [1]. Of note, a recent systematic review showed that approximately 30 cases are needed to achieve reproducible results for both open KT (OKT) and RAKT [2], highlighting that expertise in OKT and robotic urologic surgery could reduce the learning curve for RAKT.

Unfortunately, despite recent progress in proficiency-based curricula [3–5], surgical training in RAKT is still unstructured and not grounded into formal credentialing courses including simulation, dry/wet lab facilities, and modular training with animal models [3–5].

In this scenario, offering training opportunities to surgeons interested in gaining proficiency in RAKT could provide them the RAKT-specific skillset needed for a “*safer*” learning curve within more structured curricula.

In this manuscript, we report our experience with the training course on RAKT, developed by the European Association of Urology (EAU) Robotic Urology Section (ERUS) Robot-assisted Kidney Transplantation working group at the EAU Skills Center in Orsi Academy (Melle, Belgium), focusing on the value of a step-wise approach and porcine models to observe the performance of RAKT.

## Materials and methods

The hands-on 3-day course is carried out since 2016 three/four times/year and is led by expert proctors with an extensive experience in RAKT. The course allows participation of maximum eight trainees/session, to ensure one proctor for every two trainees. To maximize the course’s effectiveness, a solid knowledge of robot-assisted surgery and OKT would be desirable. All the practical sessions are performed using the da Vinci Xi, X, or Si platforms (Intuitive, Sunnyvale, USA).

**Fig. 1:**
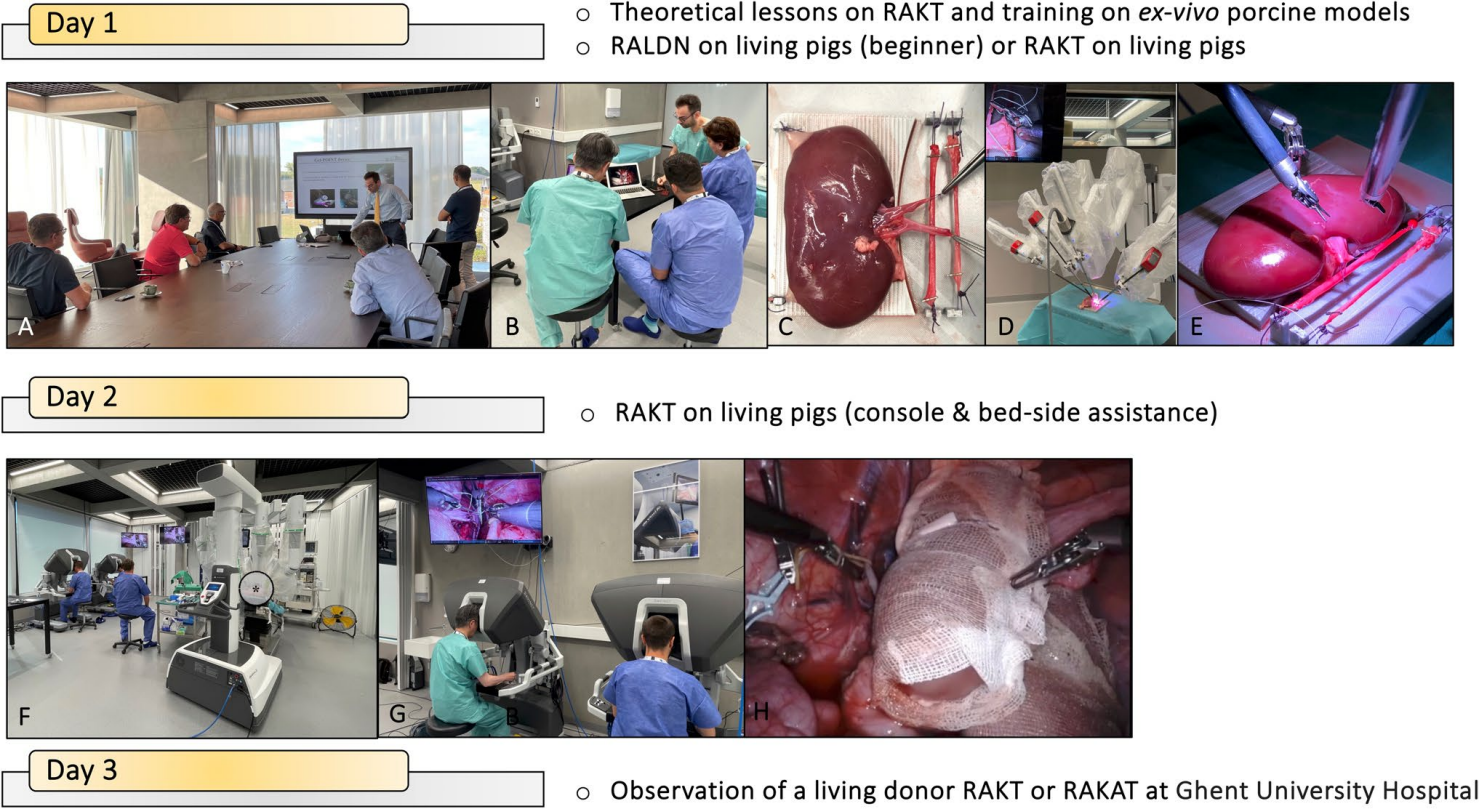
Structure of the course on robot-assisted kidney transplantation performed at the EAU Skills Center in Orsi Academy. The hands-on training 3-day course is carried out at ORSI academy. During the first day, a brief theoretical lesson on vascular anastomosis is carried out, illustrating the peculiarities of RAKT (**A**, **B**). Subsequently, on an ex-vivo porcine kidney model specifically prepared for the task, the trainees perform vascular anastomosis (**C**–**E**). During the second day, the participants perform a complete RAKT on a living porcine model. Each workstation (with a porcine model) is dedicated for two trainees. During all the steps, one trainee operates at the console and the other one stays at the table, to optimize his/her table-assistance skills (**F**–**H**). Finally, during the last day, trainees are invited to assist a real living-donor RAKT or auto-transplantation at the University Hospital of Ghent performed by one of the proctors.

The overview of the course structure is shown in Fig. 1 and includes the following activities:

- *Day 1*: the first day includes a theoretical lesson on vascular anastomoses, illustrating the peculiarities of this pivotal step in the RAKT setting (Fig. 1A, B). Subsequently, the trainees attempt to replicate these principles on an ex-vivo porcine kidney model specifically prepared for the task (Fig. 1C–E). Then, according to the trainee’s skills and experience, a robot-assisted living donor nephrectomy or RAKT in the living porcine model is carried out (as both first surgeons at the dual console, tutored by an expert proctor, and as bed-aside assistants);
- *Day 2:* the trainees attempt to perform all steps of RAKT on the living porcine model (Fig. 1F–H). Each workstation (with a porcine model) is designed for two trainees. As shown in the Supplementary Video, training of RAKT mirrors all the steps that are routinely performed in real-life practice according to the Vattikuti-Medanta technique [6–8], with few technical nuances according to the swine anatomy as well as the proctors’ preference/experience [9, 10] (Figs. 3, 4, 5, 6).
- *Day 3*: after the hands-on training sessions, the participants are invited to assist a real living-donor RAKT or auto-transplantation at the University Hospital of Ghent performed by one of the proctors (K. D.).

## Results

**Fig. 2:**
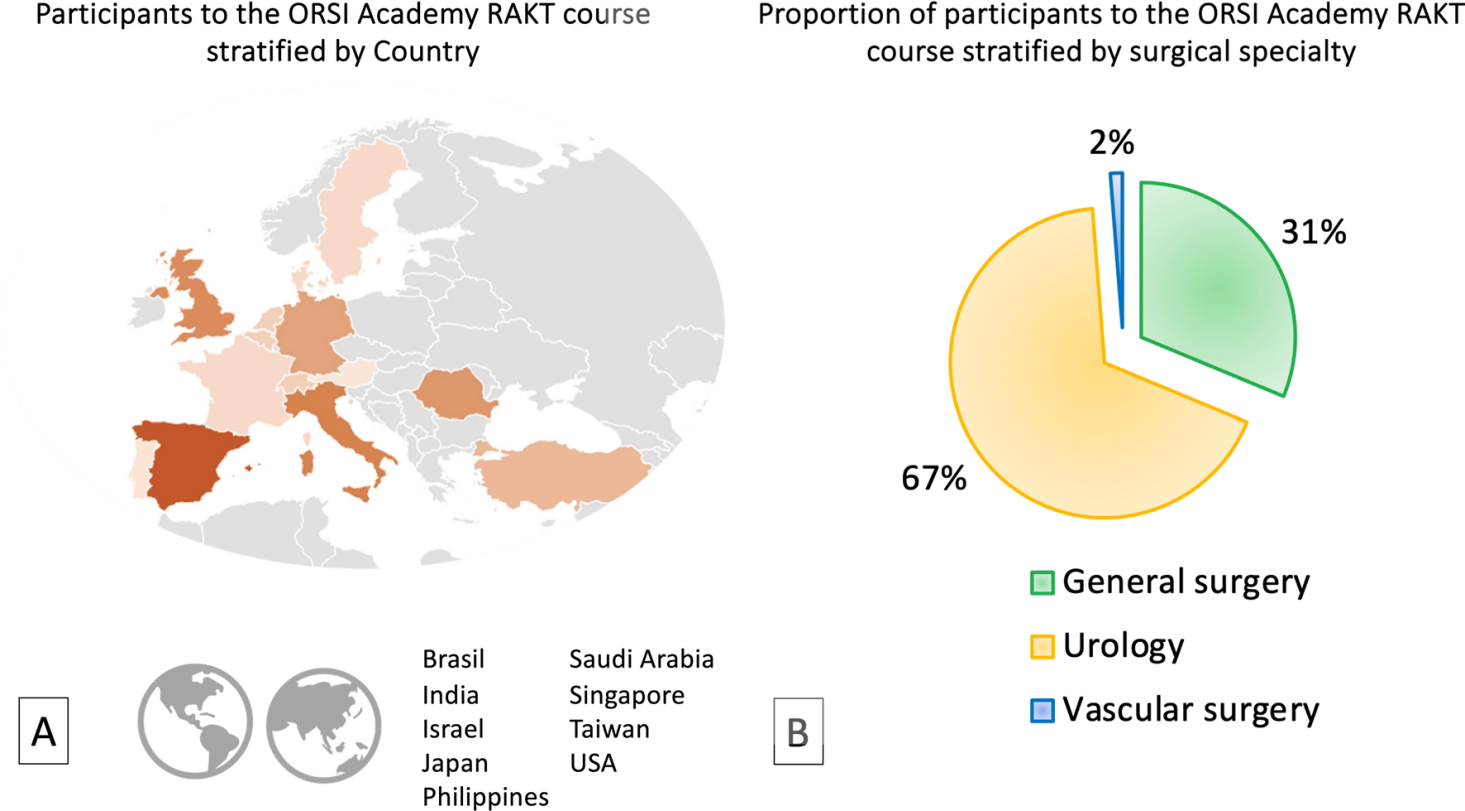
Graphical overview of the participants to the course on robot-assisted kidney transplantation performed at the EAU Skills Center in Orsi Academy, stratified by country (**A**) and surgical specialty (**B**). Overall, 78 surgeons participated in the RAKT course. Among these, Spain (16), the United Kingdom (11), and Italy (9) showed the higher number of participants. In addition, 54/78 (68%) trainees were urologists, while 25/78 (31%) were general surgeons and 1/78 (1%) was a vascular surgeon

From January 2016 to July 2023, 87 surgeons from 23 countries (of which 36% from extra-European countries) participated in the RAKT course performed at the EAU Skills Center in Orsi Academy. Of these, 58/87 (67%) were urologists, while 27/87 (31%) were general surgeons and 2/87 (2%) were vascular surgeons (Fig. 2). To date, 18 participants (20.6%) are actively involved in RAKT programs at institutions included in the European Association of Urology (EAU) Robotic Urology Section (ERUS)–RAKT network.

## Discussion

Kidney transplantation is a multidisciplinary and challenging field that has resisted innovation from a surgical standpoint for the last 50 years [11]. After the development and the standardization of the surgical technique following the IDEAL recommendations [12], RAKT has been implemented in several referral centers worldwide and increasingly performed in both living- and deceased-donor settings [13, 14].

Considering the advantages of robotic platforms and the current relevance of simulation in surgical training, several proficiency-based progression training curricula have been proposed and validated during the last years for several urological procedures [3].

**Fig. 3:**
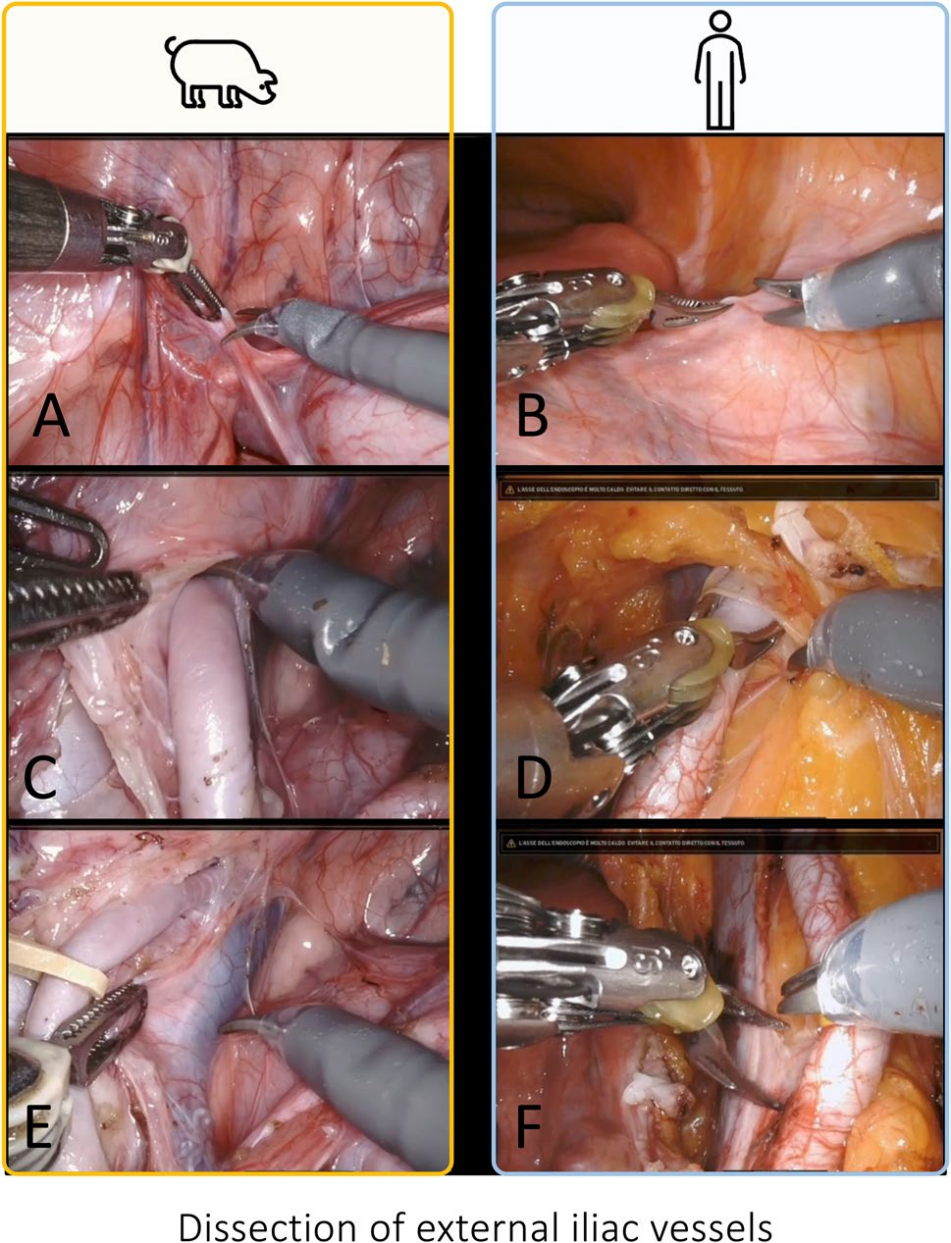
Intraoperative snapshots showing the isolation of external iliac vessels during a RAKT on a porcine model and on a human being. The first phase is the identification of iliac vessels and the dissection of the peritoneum above iliac vessels (**A**, **B**). Then, the isolation and dissection of the external iliac artery from the lymphatic tissue is carried out (**C**, **D**). Finally, the isolation and dissection of the external iliac vein from the lymphatic tissue is completed (**E**, **F**)

**Fig. 4:**
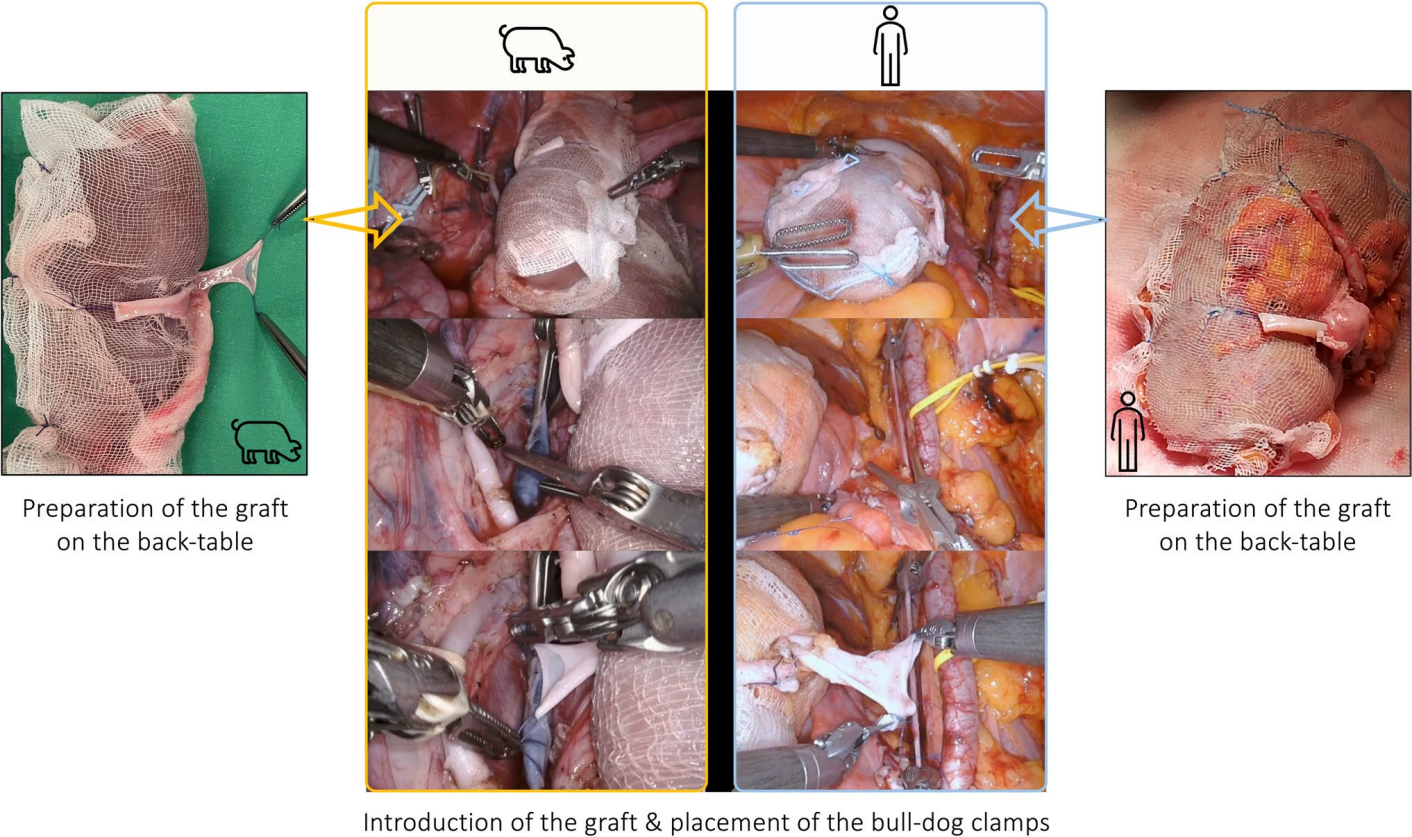
Intraoperative snapshots showing the graft preparation during bench surgery and the introduction of the graft into recipients’ abdomen during a RAKT on a porcine model and on a human being. On the left side, the graft preparation of porcine kidney and its management during vascular anastomosis during RAKT is shown. To the right side, intraoperative snapshots of the same surgical steps are reported

**Fig. 5:**
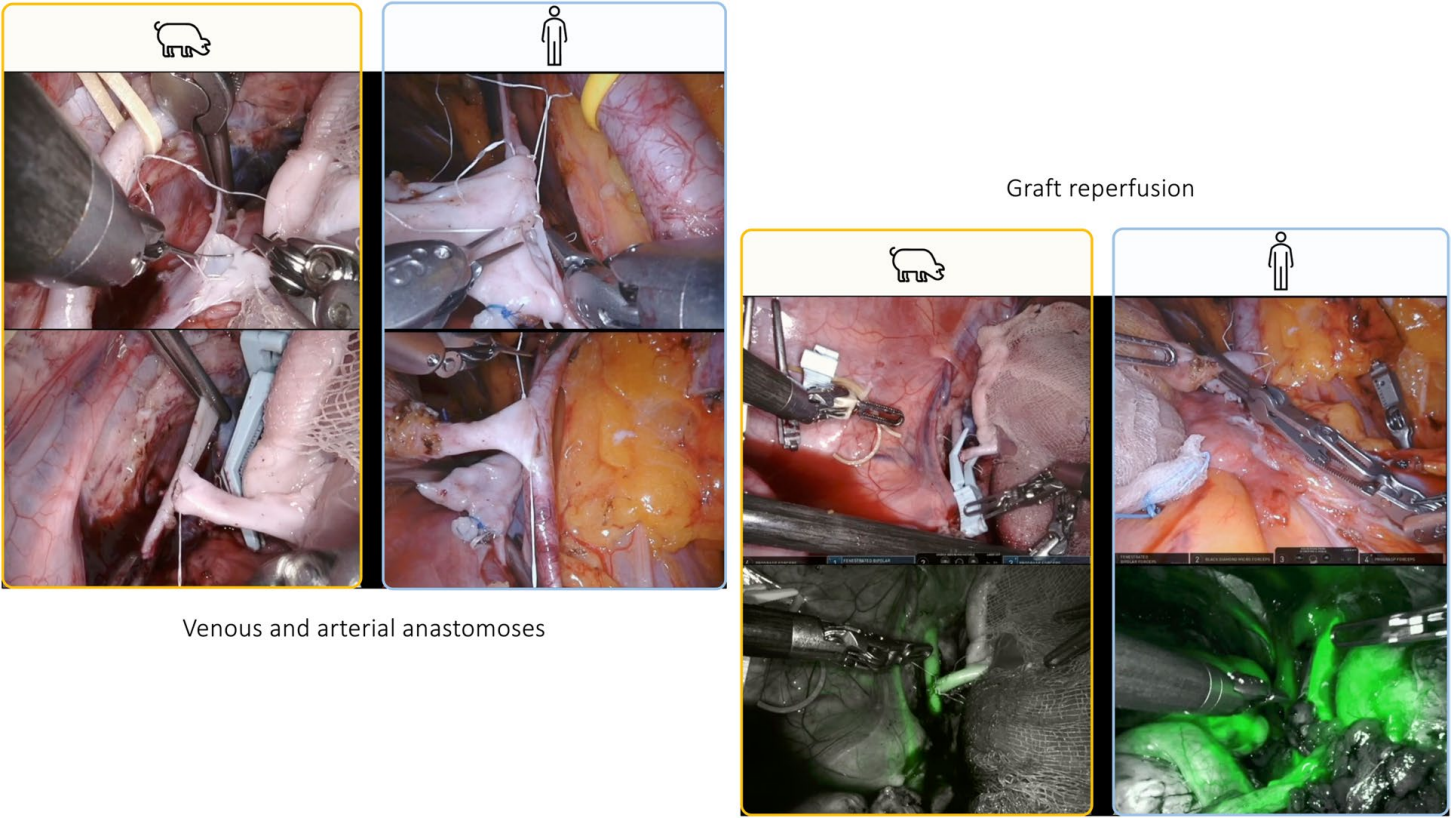
Intraoperative snapshots showing the performance of venous and arterial anastomoses and graft reperfusion during a RAKT on a porcine model and a human being. On the left side, the performance of end-to-side renal anastomoses between the graft renal vein and the external iliac vessels using a running suture are shown. On the right side, after vascular anastomoses, intraoperative snapshots of the graft reperfusion (with or without indocyanine green) are reported

**Fig. 6:**
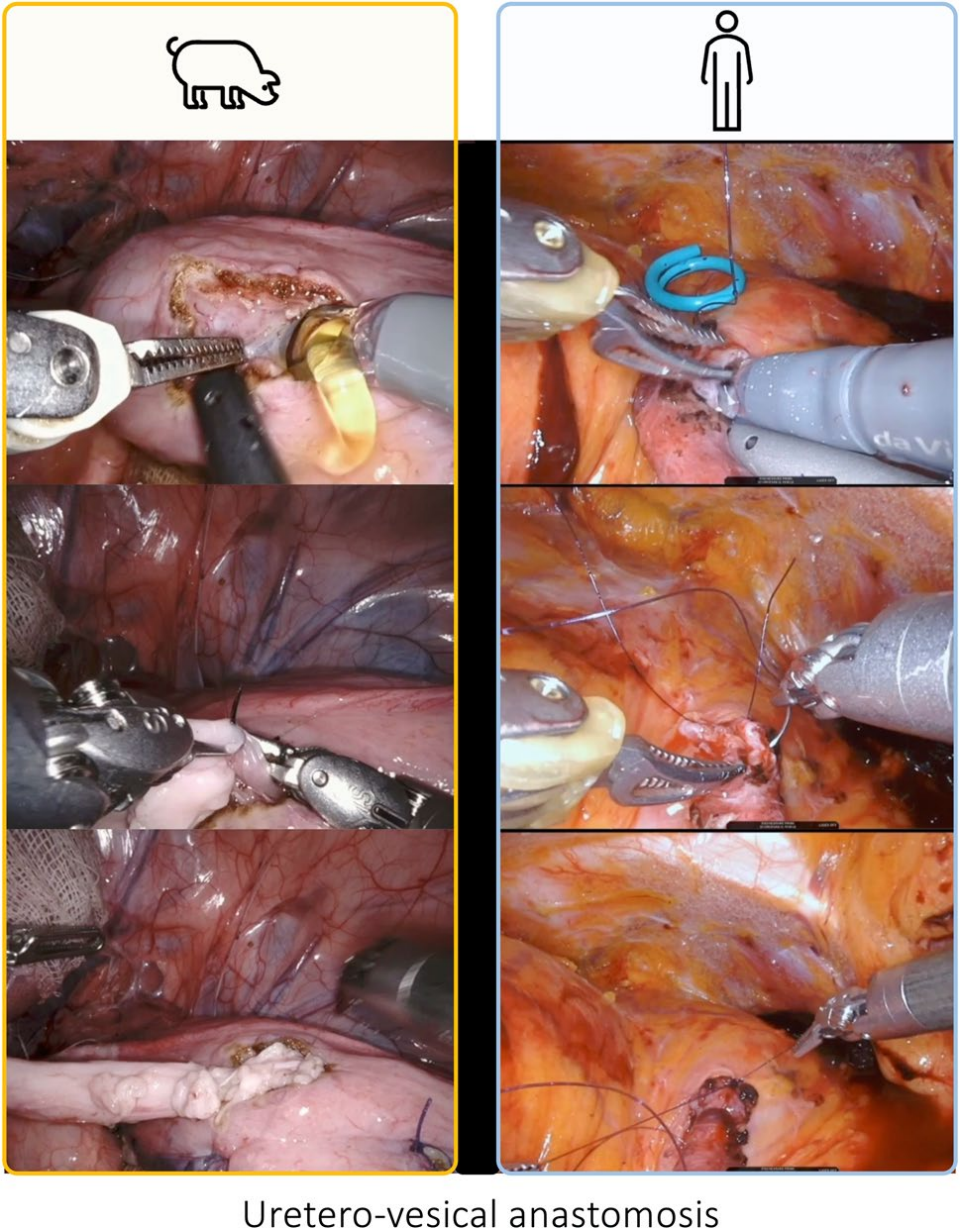
Intraoperative snapshots showing the uretero-vesical anastomosis during a RAKT on a porcine model and a human being. The uretero-vesical anastomosis was performed according to a modified Lich–Gregoir technique using a 4–0 polydiaxone suture. In humans, a 6-Fr, 16-cm double-J stent, introduced through the assistant port, is placed into the ureter before completing the anastomosis. During this phase, developing an adequate detrusor tunnel was relevant to provide an anti-reflux mechanism

In this scenario, while there is still lack of structured, validated training curricula for RAKT, the EAU Skills Center at Orsi Academy offers the first hands-on comprehensive course specifically designed to provide a safe environment where transplant surgeons (urologists, general surgeons, and vascular surgeons, Fig. 2) can be trained in the technical skills required to complete vascular anastomoses and all other RAKT-specific steps, being the final aim to maximize patient safety while introducing a RAKT program in clinical practice. Of note, trainees can effectively perform all surgical steps of the procedure (Figs. 3, 4, 5, 6), mirroring the same technique employed at referral transplant centers worldwide [7]. The course relies on the apprenticeship model [3], keeping a ratio between trainer and trainees of 1:2 to provide a strong relationship between them, with a gradual increase in complexity and surgical skill acquisition. In addition, the porcine model allows trainees to experience a large spectrum of unexpected intraoperative scenarios (i.e., adverse events, instruments’ malfunction, etc.); managing such events (under the guidance of expert proctors) may further support the surgeons’ learning process and might be key to minimize the risk of (or avoid) complications in future real-life practice. In this regard, an ideal trainee may be a surgeon with previous exposure in OKT and sufficient experience in robotic surgery to effectively acquire the theoretical and practical skillset required to start a learning curve in RAKT.

The RAKT course performed at the EAU Skills Center in Orsi Academy is not devoid of limitations. First, the course requires a registration fee, which is lower for EAU members (https://www.orsi-online.com). Second, the course does not provide training in basic robotic or KT skills, which should be considered as a prerequisite for effective training in RAKT. Third, detailed data concerning the performance of surgeons during RAKT training are not collected prospectively, thereby hindering the assessment of the course’s potential impact on the trainees’ learning curve using standardized and previously validated metrics. Finally, the training course is mainly focused on the console surgeon rather than the entire operating theater team (scrub nurse, bed-side assistant, etc.), whose knowledge, skills, and motivation are key for successful integration of RAKT into a running KT program. Yet, during the course, the trainees can get clinically relevant insights on the operating room setup for RAKT as well as on the specific tasks that need to be accomplished by each member of the team, including scrub nurses and bed-side assistants.

In future, the course could be further improved by adding online pre-course theoretical training materials, specific exercises for vascular anastomoses on 3D-printed simulators using disposable vessels [15] before modular training in the live porcine model, as well as specific training in non-technical skills applied to RAKT, given their value for a successful RAKT program. Furthermore, the course could provide specific step-by-step training to efficiently manage the additional technical and logistical challenges of RAKT from deceased donors [13]. Lastly, the clinical impact of the course could be enhanced harnessing the power of augmented reality-enhanced tele-proctoring platforms to offer trainees an opportunity to be tutored after the course during real-life RAKT until full proficiency is achieved (according to standardized metrics [3]).

## Conclusion

Leveraging the potential of simulation, wet-lab training, live porcine models, and experienced proctors, the RAKT course performed at the EAU Skills Center in Orsi Academy represents the first structured teaching effort aiming to offer surgeons a full immersion in RAKT to train the core technical skills required for safe development of a RAKT program at their own institution within the umbrella of the ERUS RAKT working group.

## Supplementary Information

Below is the link to the electronic supplementary material.

Supplementary Video. The ORSI Academy structured course on robot-assisted kidney transplantation (RAKT), with focus on the similarities between the technique in human patients and porcine models. Supplementary file1 (MP4 116,664 KB)

## References

[1] Musquera M, Peri L, Ajami T et al (2021) Robot-assisted kidney transplantation: update from the European robotic urology section (ERUS) series. BJU Int 127(2):222–228 32770633 10.1111/bju.15199

[2] Pecoraro A, Andras I, Boissier R, EAU Young Academic Urologists (YAU) kidney transplantation working group et al (2022) The learning curve for open and minimally-invasive kidney transplantation: a systematic review. Minerva Urol Nephrol 74(6):669–679 35622352 10.23736/S2724-6051.22.04909-6

[3] Collins JW, Levy J, Stefanidis D et al (2019) Utilising the Delphi process to develop a proficiency-based progression train-the-trainer course for robotic surgery training. Eur Urol 75(5):775–785 30665812 10.1016/j.eururo.2018.12.044

[4] Larcher A, De Naeyer G, Turri F, ERUS Educational Working Group and the Young Academic Urologist Working Group on Robot-assisted Surgery et al (2019) The erus curriculum for robot-assisted partial nephrectomy: structure definition and pilot clinical validation. Eur Urol 75(6):1023–1031 30979635 10.1016/j.eururo.2019.02.031

[5] Dell’Oglio P, Turri F, Larcher A, ERUS Educational Working Group and the YAU Working Group on Robot-assisted Surgery et al (2022) Definition of a structured training curriculum for robot-assisted radical cystectomy with intracorporeal ileal conduit in male patients: a Delphi consensus study led by the ERUS educational board. Eur Urol Focus 8(1):160–164 33402314 10.1016/j.euf.2020.12.015 PMC9435953

[6] Menon M, Abaza R, Sood A et al (2014) Robotic kidney transplantation with regional hypothermia: evolution of a novel procedure utilizing the IDEAL guidelines (IDEAL phase 0 and 1). Eur Urol 65(5):1001–1009 24287316 10.1016/j.eururo.2013.11.011

[7] Menon M, Sood A, Bhandari M et al (2014) Robotic kidney transplantation with regional hypothermia: a step-by-step description of the Vattikuti urology institute-Medanta technique (IDEAL phase 2a). Eur Urol 65(5):991–1000 24388099 10.1016/j.eururo.2013.12.006

[8] Sood A, Ghani KR, Ahlawat R et al (2014) Application of the statistical process control method for prospective patient safety monitoring during the learning phase: robotic kidney transplantation with regional hypothermia (IDEAL phase 2a-b). Eur Urol 66(2):371–378 24631408 10.1016/j.eururo.2014.02.055

[9] Li Marzi V, Pecoraro A, Gallo ML et al (2022) Robot-assisted kidney transplantation: Is it getting ready for prime time? World J Transplant 12(7):163–174 36051450 10.5500/wjt.v12.i7.163 PMC9331411

[10] Breda A, Territo A, Gausa L et al (2018) Robot-assisted kidney transplantation: the European experience. Eur Urol 73(2):273–281. 10.1016/j.eururo.2017.08.028 28916408 10.1016/j.eururo.2017.08.028

[11] Breda A, Budde K, Figueiredo A et al (2023) EAU Guidelines on renal transplantation. Edn. presented at the EAU Annual Congress Milan. Arnhem: EAU Guidelines Office

[12] McCulloch P, Altman DG, Campbell WB et al (2009) No surgical innovation without evaluation: the IDEAL recommendations. Lancet 374(9695):1105–1112 19782876 10.1016/S0140-6736(09)61116-8

[13] Campi R, Pecoraro A, Li Marzi V et al (2022) Robotic versus open kidney transplantation from deceased donors: a prospective observational study. Eur Urol Open Sci 1(39):36–46 10.1016/j.euros.2022.03.007 PMC9068739 35528789

[14] Pecoraro A, Li Marzi V, Sessa F, EAU Young Academic Urologists Working Group on Kidney Transplantation et al (2022) Urologists and kidney transplantation: the first European census. Eur Urol 82(3):336–337 35717360 10.1016/j.eururo.2022.05.032

[15] Campi R, Pecoraro A, Vignolini G, European Association of Urology EAU Young Academic Urologists Kidney Transplantation working group the EAU Robotic Urology Section Robot-assisted Kidney Transplantation Working Group et al (2023) The first entirely 3d-printed training model for robot-assisted kidney transplantation: the RAKT box. Eur Urol Open Sci 2(53):98–105 10.1016/j.euros.2023.05.012 PMC10251129 37304228

